# Molecular Targets in Precision Chemoprevention of Colorectal Cancer: An Update from Pre-Clinical to Clinical Trials

**DOI:** 10.3390/ijms21249609

**Published:** 2020-12-17

**Authors:** Nagendra S. Yarla, Venkateshwar Madka, Gopal Pathuri, Chinthalapally V. Rao

**Affiliations:** 1 Center for Cancer Prevention and Drug Development, Medical Oncology, Department of Medicine, Stephenson Cancer Center, University of Oklahoma Health Sciences Center, Oklahoma City, OK 73104, USA; Nagendra-Yarla@ouhsc.edu (N.S.Y.); Venkateshwar-Madka@ouhsc.edu (V.M.); Gopal-Pathuri@ouhsc.edu (G.P.); 2 VA Medical Center, Oklahoma City, OK 73104, USA

**Keywords:** colorectal cancer, biomarkers, precision prevention, molecular targets

## Abstract

Colorectal cancer (CRC) is one of the leading causes of cancer deaths worldwide. The initiation and progression of CRC is a multi-step process that proceeds via precursor lesions to carcinoma, with each stage characterized by its distinct molecular and tissue microenvironment changes. Precursor lesions of CRC, aberrant crypt foci, and adenoma exhibit drastic changes in genetic, transcriptomic, and proteomic profiles compared to normal tissue. The identification of these changes is essential and provides further validation as an initiator or promoter of CRC and, more so, as lesion-specific druggable molecular targets for the precision chemoprevention of CRC. Mutated/dysregulated signaling (*adenomatous polyposis coli*, β-catenin, epidermal growth factor receptor, *V-Ki-ras2 Kirsten rat sarcoma viral oncogene homolog* (*KRAS*), *tumor protein53*, Akt, etc.), inflammatory (cyclooxygenase-2, microsomal prostaglandin E synthase-1, inducible nitric oxide synthase, and other pro-inflammatory mediators), and metabolic/growth factor (fatty acid synthase, β-Hydroxy β-methylglutaryl-CoA reductase, and ornithine decarboxylase) related targets are some of the well-characterized molecular targets in the precision chemoprevention of CRC. In this review, we discuss precursor-lesion specific targets of CRC and the current status of pre-clinical studies regarding clinical interventions and combinations for better efficacy and safety toward future precision clinical chemoprevention. In addition, we provide a brief discussion on the usefulness of secondary precision chemopreventive targets for tertiary precision chemoprevention to improve the disease-free and overall survival of advanced stage CRC patients.

## 1. Introduction

Colorectal cancer (CRC) is one of the leading causes of cancer-related deaths worldwide. CRC can be preventable with early diagnosis and can sometimes lead to a complete cure. CRC is a major public health issue worldwide, with an estimated 861,000 deaths and 1.8 million new cases annually [1]. In the United States (US), an estimated 147,950 cases and 53,200 people will die from CRC in 2020 [1].

Most of the CRC cases (≈55–70%) are sporadic and about 30% are linked to genetic disorders, which are mostly related to the increased risk from hereditary polyposis syndromes or hereditary nonpolyposis colon cancer (Lynch syndrome) [1]. Familial adenomatous polyposis (FAP) accounts for about 1% of all CRC [1]. The sporadic and non-hereditary CRC linked to environmental factors, including diet, weight, food-borne mutagens, intestinal commensals, and chronic intestinal inflammation [1,2]. Most of the sporadic cases are detected at advanced stages of CRC, which makes it difficult to treat them. This gained attention by clinicians and researchers to identify novel biomarkers/targets for detection as well as precision chemoprevention of CRC.

The precision prevention of CRC is tailoring treatment recommendations for each individual patient based on their genetic basis and profiling of related biomarkers. The aim of precision prevention is to either increase the efficacy of treatment or reduce the side effects by selecting the appropriate treatment based on the tumor biomarker. The identification and development of novel biomarkers/targets is crucial to the future of precision oncology.

The initiation of CRC driven by loss of homeostasis of the epithelium of the intestine and mutations of key genes (for an example: *APC*) leads to abnormal crypt growth, leading to aberrant crypt foci (ACF) and further activation of pro-inflammatory mediators. The dysregulation of several signaling pathways within the ACF microenvironment leads to adenoma formation [2]. Furthermore, several mutations, importantly, *KRAS*, *SMAD*, and *TP53,* appear to be altered and promote adenoma to adenocarcinoma and metastasis. Moreover, genomic instability is a crucial feature in CRC initiation and development, which is mainly categorized into the chromosomal instability (≈85%) and microsatellite instability (≈15%) pathways. Chromosome instability is associated with several frequently mutated genes (*APC*, *TP53*, *KRAS*, *TGF-β* and others) of CRC during initiation and progression [3].

The review article updates the current knowledge in various biomarkers and molecular targets for the precision prevention/treatment of CRC. It describes the expression profile and the role of several markers at various stages of CRC development as evident from and supported by pre-clinical and clinical studies. Furthermore, the potential utility of some of the selected biomarkers and their targeted agents for personalized CRC chemoprevention/therapy are also reviewed.

## 2. Lesions-Specific Molecular Targets in CRC

### 2.1. Mutated and Dysregulated Signaling Precision-Molecular Targets

#### 2.1.1. APC/β-Catenin

Adenomatous polyposis coli (*APC*), a gatekeeper tumor suppressor gene, is the most commonly mutated gene in familial adenomatous polyposis (FAP) that develops many colonic polyps and has the highest risk of developing colonic cancers at an early age (<35 years) [2,4,5]. In addition, the APC gene is mutated in more than 80% of patients with sporadic CRC. Dysregulation of the Wnt/APC/β-catenin pathway is associated with *APC* mutation [2,4]. One of major functions of the APC protein is the regulation of β-catenin by its degradation and maintenance of the homeostasis of normal colon epithelial cell growth. Mutations in the *APC* gene lead to a functionally inactive, oncogenic truncated APC protein, leading to the nuclear translocation of β-catenin and induction of genes responsible for the carcinogenesis of CRC [2,4]. Clinical data suggest that CRC correlates with increased nuclear levels of β-catenin phosphorylated at serine 552 and bound with TCF4 in nucleus (Table 1) [6]. APC/β-catenin mutations were found in precursor lesions (ACF and mucin depleted foci (MDF)) and adenoma in both humans and rodents by PCR amplification and sequencing of their DNA [7]. Some studies show that an uncontrolled nuclear accumulation of β-catenin in intestinal epithelial cells in pre-neoplastic lesions is a prerequisite for the progression of tumor growth [8]. Several small molecule agents that target the Wnt/β-catenin signaling pathway are developed, and some are in various stages of their pre-clinical and clinical development for CRC. However, no drug that targets this pathway has entered into clinical practice [9]. Overall, studies demonstrated that APC/β-catenin is a well validated target for CRC prevention and warranted detailed studies to develop inhibitors of this pathway as chemopreventive agents for CRC prevention.

#### 2.1.2. KRAS/EGFR

**Figure 1:**
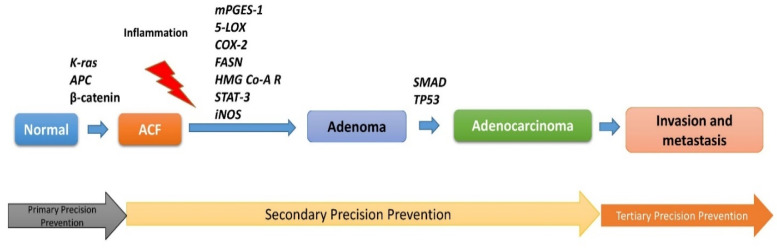
Important molecular targets during colorectal tumorigenesis and progression.

The epidermal growth factor receptor (EGFR)/V-Ki-ras2 Kirsten rat sarcoma viral oncogene homolog (KRAS) pathway plays a key role in CRC initiation and progression [10]. The KRAS gene is the most frequently mutated gene in CRC and is associated with the modulation of several downstream effectors to include: RAF/MEK/ERK, PTEN-PI3K-AKT-mTOR, RalGDS/p38MAPK, and Rac/Rho during tumorigenesis and the tumor progression of CRC [11]. The *KRAS* mutation is more frequent in ACF and colon tumors (Table 1; Figure 1) [5,12,13]. Approximately 40% of colon cancers are positive for mutations in *KRAS* [12,13]. Takahashi et al. [14] reported that *KRAS* was mutated in ACF, adenoma, and adenocarcinomas in AOM-treated colons of rats. *KRAS* mutations are more common in female patients with CRC than in male patients with CRC [15].

*KRAS* mutations are associated with a lack of response to anti-EGFR targeted therapies for CRC patients [15]. This is because *KRAS* amplification may also be responsible for primary resistance to EGFR inhibitors [16]. Therefore, oncogenic KRAS pathways are key targets for CRC prevention/therapy. However, detailed investigations are still required to demonstrate the clear role of the EGFR pathway in colorectal tumorigenesis and progression.

#### 2.1.3. TGF-β1/SMAD

TGF-β1 plays an important role in controlling gut inflammation in relation to the continuous stimulation of the intestinal microbiota. SMAD4 is a common mediator of the TGF-1β signaling pathway. Tsushima et al. [17] reported high levels of TGF-β1 in patients with colorectal cancer compared to healthy individuals (Table 1; Figure 1). *SMAD4* haploinsufficiency is associated with an increased susceptibility to bowel inflammation with variable penetrance in association with the colonic mucosal microbiota [18]. TGF-β1 suppressed the expression of pro-inflammatory markers in the colon epithelium, and the loss of its downstream mediator, *SMAD4*, is associated with the initiation of inflammation-driven colon cancer and is identified as a tumor suppressor. Loss of *SMAD4* expression is observed in 48% of human colitis-associated carcinoma samples as compared with 19% of sporadic colorectal carcinomas [19]. Hence, these studies suggested that TGF-β1/SMAD4 is a novel biomarker and target for the prevention of CRC.

#### 2.1.4. *TP53*

**Figure 2:**
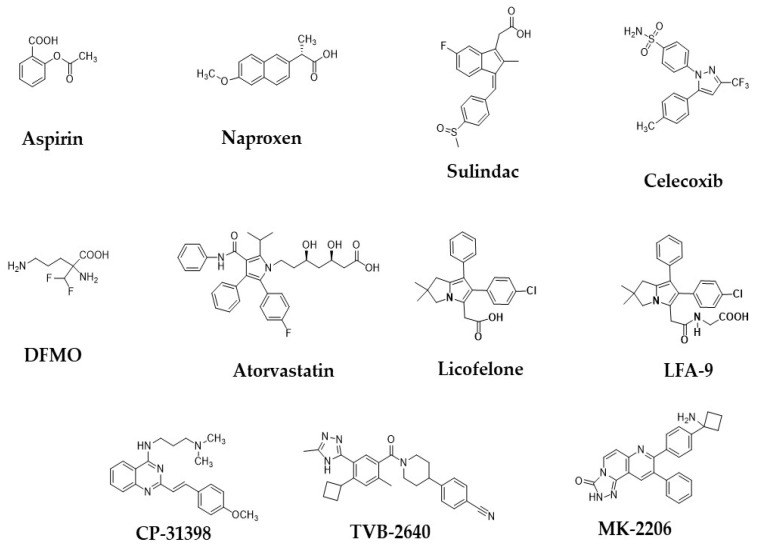
Chemical structures of some of the important precision chemopreventive agents for colorectal cancer prevention and treatment by inhibiting or modulating the various key targets.

*TP53* is a tumor suppressor gene, and the mutation of this gene commonly occurs in approximately 40–50% of sporadic CRC [20]. The status of *p53* mutation is closely related to the progression and outcome of sporadic CRC [20]. *TP53* mutations are most commonly found in adenoma to adenocarcinoma stages of CRC (Table 1; Figure 1) [21]. Previous studies suggested that p53 protein accumulation and *p53* gene mutations were not found in ACF [22,23]. Chang et al. [24] reported that the loss of p53 enhanced the induction of colitis-associated CRC by dextran sulfate sodium. Recently, Alpert et al. [25] found that *TP53* mutations are common in inflammatory bowel disease-associated colon cancer, while the frequency of *APC* and *KRAS* mutations was significantly lower than in sporadic CRC (Table 1) [25,26]. Hence, the authors concluded that *TP53* is a novel target for IBD-CRC. Previously, we reported that CP-31398 exhibited significant chemopreventive activity in a pre-clinical animal model (Figure 2) [27]. These pre-clinical studies warranted clinical studies on the combinational use of CP-31398 and celecoxib for the prevention and therapy of CRC. Overall, studies suggest that *TP53* is a promising marker for precision prevention and therapy of CRC.

#### 2.1.5. AKT

AKT pathway has been investigated extensively in CRC initiation and progression [28,29]. The lipid product of class I PI3Ks activates the downstream kinase AKT (AKT1, AKT2, AKT3), which is further involved in the pathophysiology of CRC tumorigenesis [28,29]. Clinical and pre-clinical evidence suggests that an overexpression of AKT is found in tumors of CRC as compared to normal tissues. Phosphorylated-AKT was high adenocarcinoma compared with adenoma and normal colonic mucosa [29]. Roy et al. [30] reported that AKT is overexpressed in adenomas of sporadic CRC but not in normal colonic mucosa and/or hyperplastic polyps. A selective inhibitor of AKT MK-2206 is under clinical evaluation with combination of other drugs for treatment of the patients with CRC (Figure 2) [31]. Thus, the AKT pathway has been considered as a target in initial and later stages of CRC.

### 2.2. Inflammatory Targets: ACF Progression to Adenoma

#### 2.2.1. Cyclooxygenase-2

Cyclooxygenases (COX-1/2) are key enzymes in eicosanoids biosynthesis that catalyze arachidonic acid to PHG_2_. COX-1 is a constitutive enzyme and involved in normal physiological functions, while COX-2 is an inducible enzyme and plays a key role in the pathophysiology of inflammation and carcinogenesis. In rodent and human colonic tumors, COX-2 is overly expressed as compared to COX-1 [32]. The role of COX-2 in CRC is well established, and it is a validated target for adenoma prevention in both pre-clinical and clinical settings [33]. COX-2 expression is associated with multi-crypt-ACF and MDF, whereas no expression in normal crypts of the colon was observed [34]. The activated COX-2 pathway in ACF may be involved in its further progression to adenoma [32]. COX-2 is predominantly overexpressed in adenoma compared to adjacent normal colorectal mucosa (Table 1 and Figure 1) [35,36]. Polymorphism in the *COX-2* gene (-765G/C region) was associated with an increased risk of CRC by several folds [37]. Nonsteroidal anti-inflammatory drugs, or NSAIDs (aspirin, naproxen, sulindac) and COX-2 selective inhibitors COXibs, such as celecoxib, are widely used in the clinic for CRC prevention (Figure 2) [38,39,40,41,42,43,44]. Chan et al. [42] found that the regular use of aspirin reduced the risk of CRC patients with an overexpression of COX-2. In a prospective and case-control study, the use of aspirin/NSAID appeared to lower the risk of COX-2-positive cancers, particularly among individuals with high levels of a circulatory inflammatory biomarker macrophage inhibitory cytokine-1 [43]. Hence, COX-2 is a novel target/biomarker in CRC, and its inhibitors have been widely used for the prevention and therapy of CRC.

#### 2.2.2. Microsomal Prostaglandin E Synthase-1

Microsomal prostaglandin E synthase-1 (mPGES-1) is an inducible enzyme in inflammatory and oncologic conditions among other constitutive isoforms i.e., cytosolic PGES and mPGES-2, which specifically acts on PGH_2_ released by the activity of COX-2 and converts to PGE_2_ (Figure 1). mPGES-1 was reported to be overexpressed in the large ACF and adenomas and more so, in the adenocarcinomas, compared to the matched normal tissues (Table 1) [45,46,47,48,74]. The genetic deletion of mPGES-1 suppresses intestinal tumorigenesis in *Apc*D^14/+^ mice [45]. The deletion of mPGES-1 reduced the size and number of pre-neoplastic aberrant crypt foci (ACF) and blocked β-catenin nuclear translocation in carcinogen-induced ACF [45]. It also caused up to an 80% decrease in tumor multiplicity and up to a 90% reduction in tumor load in the distal colon of AOM (carcinogen)-treated mice compared to wild-type mice [74]. Moreover, a mechanistically enzymatic inhibition of mPGES-1 resulted in the suppression of PGE_2_ production and sparing the prostacyclin I_2_ and thromboxane B_2_, which circumvent the unwanted side effects associated with the inhibition of COX-2 activity [39]. Several mPGES-1 inhibitors have been developed as chemopreventive agents, and some of them are under various stages of their pre-clinical and clinical evaluation with a better safety profile [39]. Therefore, mPGES-1 is a novel and precision target for the prevention of CRC with better safety.

#### 2.2.3. 5-Lipoxygenase

The lipoxygenase (5-LOX) involves the chronic inflammation and carcinogenesis of CRC. Accumulating evidence suggests a potential role of 5-LOX and its products in early and advanced stages of CRC carcinogenesis [50,51,75]. 5-LOX expression levels are higher in patients with CRC than healthy individuals and have found its expression levels associated with tumor initiation and progression [49,50,51]. 5-LOX inhibitors (e.g., zileuton) exhibited a chemopreventive effect in pre-clinical models of CRC [76]. Overall, studies so far suggest that 5-LOX is a precision target in CRC.

#### 2.2.4. Prostacyclin I_2_ Synthase

Prostacyclin (PG I_2_), products of prostacyclin synthase (PGIS), have been characterized as anti-inflammatory mediators and tumor suppressors. PGI_2_ levels are suppressed in CRC compared to normal colonic mucosa [61]. Cutler et al. [77] reported that the stromal production of PGI_2_ showed an anti-apoptotic effect in colonic epithelial cells. The genetic deletion of prostacyclin synthase (PGIS) enhanced ACF formation at the early stage of carcinogenesis [59,60]. These results suggest that PGIS and PGIS-derived PGI_2_ are involved in anti-carcinogenic effects. Mechanistic studies demonstrated that epigenetic inactivation of the *PTGIS* gene (hypermethylation of the PTGIS promoter) is associated with colorectal carcinogenesis [78]. The aforementioned studies suggested that PGIS is a biomarker for CRC.

#### 2.2.5. 15-Hydroxyprostaglandin Dehydrogenase

15-hydroxyprostaglandin dehydrogenase (15-PGDH) is an NAD(+)-dependent enzyme, which is involved in the degradation of PGE_2_ in a normal colon. 15-PGDH levels are significantly down-regulated in adenoma and adenocarcinoma as compared to normal colonic mucosa (Table 1) [56]. In an AOM/DSS-promoted carcinogenesis mouse model, 15-PGDH levels are low/abolished in colonic adenocarcinoma [55]. The expressions of 15-PGDH, apart from COX-2, in pre-treatment adenomas, provides predictive information in patients treated with celecoxib for the prevention of CR adenomas based on a study by Wang et al. [79]. In addition, elevated HDAC expression is correlated with the down-regulation of 15-PGDH in human colon cancers [80]. Moreover, HDAC inhibitors enhanced 15-PGDH expression in CRC cells [80]. Hence, 15-PGDH can be a predictive biomarker of CRC, and its enhancers are needed for CRC prevention.

#### 2.2.6. 15-Lipoxygenase-1

15-lipoxygenase-1 (ALOX15 or 15-LOX-1), a member of the AA pathway, is involved in the formation of lipoxins and resolvins to resolve inflammation and cancer of the colon [62,64,81]. The expression of ALOX15 was less in adenoma of patients with CRC as compared to that of healthy individuals (Table 1) [64]. Transgenic expression of ALOX15 in the intestine of mice inhibited DSS-induced colitis and chemical-induced CRC tumorigenesis [55,79]. ALOX15 inhibited several pro-inflammatory mediators, which have a key role in the promotion of colorectal cancer via an activation of chronic inflammation [64,82]. ALOX15 expression suppressed the invasion of CRC cells [83,84]. 15-LOX-1 exhibits anti-angiogenic effects through reduced VEGF levels in CRC cells [85]. 15-LOX-1 improved the tumor-suppressive effect of NSAIDs and celecoxib, and this effect is associated with its overexpression [86]. Previous studies suggested that 15-LOX-1 exhibited tumor-suppressive effects during CRC tumorigenesis and can be a biomarker for CRC.

#### 2.2.7. Inducible Nitric Oxide Synthase

Inducible nitric oxide synthase (iNOS), an inducible enzyme during inflammatory conditions, plays a key role in CRC initiation and progression. Among isoforms of nitric oxide synthase (nNOS, eNOS, and iNOS), iNOS has a key role in the pathophysiology of inflammatory and oncologic diseases, including CRC [2]. iNOS expression and activity were higher than normal in cancerous tissues of the colon (Table 1 and Figure 1) [52,53,54]. iNOS is overexpressed in precursor lesions, such as MDF [34]. The expression of iNOS was increased after the transition from hyperplastic ACF to dysplastic ACF, adenoma, and carcinoma [87]. Regular exercise prevents CRC tumorigenesis, which is partly mediated through the suppression of iNOS expression-associated inflammation [88]. Previous reports suggest that chronic inflammation and iNOS-mediated NO promotes neoplastic transformation of the colorectum, which leads to the carcinogenesis of CRC [2,89,90]. Mice with mutations in both *APC* and *iNOS* showed a reduction in adenomatous polyps in the small and large intestines compared to mice with the mutation in *APC* alone [91]. Moreover, several iNOS inhibitors showed a significant chemopreventive efficacy of CRC [92]. Hence, iNOS is a promising target in CRC prevention.

#### 2.2.8. STAT-3

Signal transducer and activator of transcription (STAT), a group of signaling molecules, is involved in various physiological functions of cell proliferation and survival. The activation STAT (-1, -3 and -5) signaling pathway plays a key role in CRC tumorigenesis and progression [2,58]. STAT3 is overexpressed in malignant tissues compared to normal based on experimental and clinical data [57,58]. Li et al. [58] reported the prognostic role of phospho-STAT3 in patients with cancers. The STAT-3 signaling pathway plays a key role in the pathophysiology of IBD and colorectal cancer along with other pro-inflammatory mediators [93]. The activation of STAT-3 involves in the growth of CRC cells [94]. Previously, our group reported that regulatory T cells promote intestinal tumorigenesis via the activation of IL-22 and STAT-3 [95]. Chen et al. [96] reported that black raspberry anthocyanins prevented carcinogen-induced CRC by targeting several oncogenic mediators, including STAT-3. The aforementioned finding suggested that STAT-3 can be a novel target in CRC.

#### 2.2.9. Chemokine Receptor 5

Chemokine receptor 5 (CCR5) is a chemokine receptor for chemokine CCR3 that plays an important role in CRC progression of CRC [96,97,98]. Löfroos et al. [98] reported that infiltrating T lymphocytes in colorectal cancers showed an overexpression of CCR5 (Table 1). CCR5-deficient mice failed to develop colon tumors in DSS/AOM-treated mice compared to wild-type mice [97]. Clinical data demonstrated that CCR5 expression in CD4 T-cells is associated with an increased risk of CRC [99]. Maraviroc, an antagonist of CCR5 and a drug for AIDS, suppressed the tumor formation of a murine CRC cell line and the growth of orthotopically injected colon cancer cells [100]. CCL5 is produced by lymphocytes and promotes tumor cell growth. In a clinical study, CCL5 antagonist showed a considerable effect in blocking CRC progression [101]. Previous studies suggest that CCR5 is novel target in CRC and its antagonist, maraviroc, can be a novel agent for CRC prevention and therapy based on future studies. Further detailed studies are warranted on CCR5 to validate as novel target in CRC at pre-clinical and clinical levels.

### 2.3. Metabolic and Growth Factor-Related Targets

#### 2.3.1. Fatty Acid Synthase

Fatty acid synthase (FASN), a lipogenic enzyme, plays a key role in the carcinogenesis and development of CRC [102]. The overexpression of FASN was found in ACF and tumors with sporadic CRC or FAP [73]. FASN was highly expressed in rectal biopsies from patients harboring colonic adenomas as well as colonic mucosa of both the AOM-treated and *APC*^pirc^ rats as compared to that of healthy tissue (Table 1) [72]. Increased FASN activity is associated with a decreased survival of patients with CRC [103,104]. FASN levels in serum are associated with different stages of colorectal cancer patients [105]. FASN inhibitors have been developed and are currently under evaluation of various stages of pre-clinical and clinical trials for CRC [103,104,105,106]. Oral FASN inhibitor (TVB-2640) entered a Phase I clinical trial (3V2640-CLIN-002) in solid tumor patients, and the study is demonstrating a favorable tolerability profile without any significant adverse events (Figure 2) [102]. Expression levels of FASN are more frequent in patients with advanced CRC [72,73]. In vitro studies confirmed that the knockdown of FASN in various CRC cell lines hindered the invasive capability of cancer cells, and these results suggested the pro-metastatic role of FASN in CRC tumorigenesis [107]. Zaytseva et al. [108] found that the overexpression of FASN is associated with advanced stages of CRC and liver metastasis. Kuchiba et al. [109] demonstrated that cellular FASN status determines a cell’s dependence on energy balance status for the malignant transformation of CRC. Chang et al. [110] reported that the blockade of FASN is associated with an inhibition of cell proliferation and induction of apoptosis. Hence, the aforementioned suggests that FASN is a novel target in CRC and its pharmacological inhibitory agents can be novel preventive and therapeutic agents for CRC.

#### 2.3.2. Ornithine Decarboxylase

Ornithine decarboxylase (ODC) is a key enzyme in the biosynthesis of polyamines, which is involved in the proliferation of cells in colorectum. ODC activity of cancer tissue or adenoma tissue of CRC was significantly higher than that of normal tissues based on pre-clinical and clinical studies [65,66,67,68]. ODC inhibitors have been developed as chemopreventive agents for CRC [111,112]. D, L-α-diflouromethylornithine (DFMO), a selective ODC inhibitor, suppressed APC-dependent intestinal tumorigenesis in mice and was found to be less toxic (Figure 2) [65,113]. In clinical trials, DFMO inhibited ODC enzyme activity and polyamine contents and exhibited a preventive efficacy of CRC [111,112]. In a randomized clinical trial, DFMO in combination with the NSAID sulindac reduced the adenoma recurrence rate among individuals with colonic adenomas when compared with placebos [110]. Previously, we evaluated different combinations of chemopreventive agents with DFMO for CRC prevention in a chemical carcinogen-induced rat CRC model [114]. DFMO in combination with rosuvastatin alone or a combination strategy showed a significant suppression of colon adenocarcinomas in carcinogen-induced CRC [114]. In another study from our group, piroxicam plus DFMO significantly inhibited colon adenocarcinoma incidence in carcinogen-induced CRC [115]. Kumar et al. [116] found that the oral supplementation of ellagic acid caused the transcriptional inactivation of ODC expression, reducing ACF proliferation and/or progression, thereby exhibiting the chemopreventive efficacy of EA against CRC. Pre-clinical and clinical studies demonstrated that D, L-α-diflouromethylornithine (DFMO), a selective ODC inhibitor, exhibited chemopreventive efficacy on CRC [111,112]. Hence, ODC can be a promising target, and its pharmacological inhibitor DFMO can be a potential chemopreventive agent for CRC.

#### 2.3.3. HMG Co-A Reductase

HMG Co-A Reductase (HMG Co-A R) is an enzyme involved in cholesterol synthesis and has a key role in the tumorigenesis of colorectal cancer. HMG Co-A R is overexpressed/high activity from normal epithelium to colorectal tumors of early or advanced stages of rodents and humans (Table 1 and Figure 1) [69,70,71]. Bengtsson et al. [71] reported that the significant associations of HMG Co-A reductase expression with expressions of cell survival markers in colorectal cancer. Inhibitors of HMG CO-A reductase are called statins, which have been widely explored as preventive and therapeutic agents for CRC [69,117]. Karagkounis et al. [118] reported that the combination of radiation and simvastatin inhibited the cell growth and viability of different CRC lines. Wei et al. [69,119] found that HDAC and HMG Co-A reductase dual inhibitory statin hydroxamic acid derivatives showed significant preventive efficacy on inflammation-driven colorectal tumorigenesis in rodent models. These studies suggest that the dual targeting of HDAC and HMG Co-A reductase can be a better strategy for the prevention of CRC. Atorvastatin, an HMG-CoA R inhibitor, suppressed intestinal tumorigenesis, and its chemopreventive efficacy was increased with low doses of celecoxib or NSAIDs in an *APC*^min\−^ mice (Figure 2) [120,121]. Previously, we reported that COX inhibitors and HMG Co-A reductase inhibitors (statins) were effective in chemopreventive efficacy than alone at a low dose [121]. In a population-based case-control study by Lipkin et al. [122], a single-nucleotide polymorphism was identified in the *HMG Co-A* gene that significantly modified the chemopreventive protective association between statins and CRC risk. Therefore, HMG-CoA R has been proven as a promising target, and its inhibitors statins exhibited promising efficacy in CRC secondary prevention.

## 3. Molecular Targets and Tertiary Precision Prevention

The importance of tertiary precision prevention has grown due to the increasing mortality and morbidity of CRC. A lack of well validated surrogate biomarker(s) at the pre-clinical and clinical level has limited the development of precision preventive strategies to improve the cancer survivors. Pre-clinical and clinical studies demonstrate that several factors with a major impact on the risk of developing CRC are also related to the cancer survival, which highlights opportunities for tertiary precision prevention. In this scenario, the role of chemoprevention in tertiary precision prevention is currently an important subject to intensive research. COXs, ODC, and HMG Co-A reductase are the most validated targets for CRC tertiary prevention, and their inhibitors (NSAIDs, COXibs, and statins) showed promising efficacy in the reduction of adenocarcinoma based on pre-clinical and clinical evaluations [40,123,124,125,126,127]. Previously, our group reported that the administration of 1500 ppm celecoxib during the progression stage also significantly suppressed the incidence and multiplicity of adenocarcinomas of the colon [126]. A meta-analysis by Li et al. [127] demonstrated that the use of aspirin after diagnosis improves CRC survival. A phase III double-blind placebo-controlled randomized trial of aspirin (80 mg given orally) once daily for five years is ongoing to evaluate five-year overall survival for stage II and III colon cancer patients and recurrence (https://clinicaltrials.gov/ct2/show/NCT02301286) [124]. mPGES-1 is expressed and correlated with a significantly worse prognosis in stage I–III patients of CRC. However, more studies are required to validate the mPGES-1 as a novel target for the safer tertiary precision prevention of CRC [125]. Overall, pre-clinical and clinical studies suggested the emerging role of several molecular targets and its inhibitors in the tertiary precision prevention of CRC.

## 4. Pre-Clinical Rodent Animal Models for Biomarkers-Based Precision Chemoprevention

Several animal models have been developed for biomarkers-based research relevant to the chemoprevention of CRC, including chemically induced CRC and genetically modified rodent models, which are useful in pre-clinical novel biomarkers identification and target-based drug development for human CRC preventive/therapeutic intervention. Among the chemically induced CRC models, the combination of azoxymethane (AOM) with the inflammatory agent dextran sodium sulfate (DSS) in rodents has proven to dramatically shorten the latency time for the induction of CRC with ACF–adenoma–carcinoma sequence that occurs in human CRC [128]. This model has been helpful in the identification of biomarkers/agents for primary, secondary, and tertiary chemoprevention of inflammation-associated and sporadic CRC. The *APC*^min/+^ mouse model is the most commonly used model related to the *APC* gene heterozygous mutation and β-catenin pathway activation, and it is helpful in the identification of biomarkers/agents for the chemoprevention of hereditary CRC [129]. Tetteh et al. [130] developed an inducible colon-specific cre enzyme mouse line for proximal tumors development with *APC*/*KRAS* mutation and aggressive carcinomas with some invasion into lymph nodes also developed upon a combined induction of oncogenic mutations of *APC*, *Kras*, *p53*, and *SMAD4* in quadruple-mutant mice. *APC*^pirc^ is a novel model in rats, which mimics the FAP of humans with pre-neoplastic polyps development in the colorectum [6,131,132]. Moreover, MDF are formed in colons of the rats during CRC initiation and associated with nuclear β-catenin accumulation (Table 1) [123,124]. The aforementioned models are widely used for the identification of novel targets and target-based agents at the pre-clinical level for the chemoprevention of CRC [131,132].

Several pre-clinical models have been developed for studies related to Lynch syndrome [133,134,135]. Homozygous *Msh2*, *Msh6*, and *Mlh1* knockout mice are cancer-prone, developing tumors in different organs including the colorectum. The disadvantage of these homozygous knockout mice is they usually die by 6 to 8 months due to aggressive lymphomas [133,134]. To overcome the early death, conditional *Msh2* knockout mice, in which intestine-specific gene inactivation is permitted, have been generated using *Villin-Cre* or *Cdx2-NLS-Cre.* These mice develop tumors that highly mimic the tumors developed by patients with Lynch syndrome [134]. Biallelic germ-line mutations in the *MUTYH* gene, which encodes for a DNA glycosylase involved in base excision repair, have been identified in patients with hereditary multiple colorectal adenoma and carcinoma. A *Mutyh*^−/−^ mouse strain was developed and their response to oxidative stress in the form of exposure to dextran sulfate sodium (DSS) was assessed [135]. The mice model development has helped to understand the base–excision–repair and inflammatory response in CRC tumorigenesis.

In addition, xenografts and orthotopic rodent animal models have been developed for CRC carcinogenesis studies and therapeutic drug development [136]. CRC stem cells appeared in the tumors of the xenograft model, but the lack of active immune system in these models is the drawback for their utility in immunomodulation studies [136]. Subcutaneous xenografts are not useful for CRC local tumor invasion and CRC metastatic research, while the orthotopic model is useful for studies related to metastasis [136]. Moreover, the molecularly annotated patient-derived xenograft “Avatar” models are providing an excellent opportunity for co-clinical trials to determine the clinical decisions, including the identification of precision-targeted therapeutic agents, for patients with CRC in a real-time manner [137].

## 5. Conclusions and Perspectives

Biomarker analyses of CRC are important in order to diagnose and develop precise target specific agents for the precision chemoprevention of CRC. Over the last decade, tremendous progress has been made by several research groups in the identification and development of biomarkers/targets for the precision prevention of CRC, which is one of the reasons for the drop in the death rate in men and women in the US. Some of the biomarkers can be surrogate endpoint markers (APC, β-catenin, HMG-CoA R, COX-2, mPGES-1, 5-LOX, TP53, KRAS, TGF-β1, AKT, and ODC) other than ACF/adenoma, which can be useful to understand the CRC development and also to evaluate the effectiveness of the agent’s preventive/therapeutic intervention.

The mutation of the APC gene and activation of β-catenin occurs in the initiation of CRC. Therefore, the APC/β-catenin pathway is a key target in the prevention of CRC at an early stage. Several inhibitors of the APC/β-catenin pathway are under pre-clinical and clinical evaluation for prevention and/or treatment of CRC. *KRAS* is one of the most commonly mutated genes in CRC, and EGFR/KRAS has been considered as an important target in the prevention of CRC. Mutations of *TP53* and *SMAD* and the activation of PI3K/AKT play an important role in the adenoma–carcinoma sequence.

There are several biomarkers of inflammation; for instance, COX-2 is the most validated target in the secondary precision prevention of CRC. The incidence of colitis and other inflammation-associated CRC has diminished from the last decades due to the use of NSAIDs. Aspirin, sulindac, naproxen, and other NSAIDs, as well as COXibs such as celecoxib, are widely used for the secondary and tertiary prevention of CRC. However, gastrointestinal, cardiovascular, and renal side effects are limiting their usage. In this scenario, combinational therapies such as COX inhibitors and other chemopreventive agents such as statins, or targeting downstream mediators of COX-2 such as mPGES-1, have been validated as a target for the safer chemoprevention of CRC. Recent findings suggest that COX-2 or mPGES-1 alone are not very effective in blockade of the inflammation due to arachidonate metabolic pathway diversion to leukotrienes production by the activation of 5-LOX. Further, COX/5-LOX inhibitors (e.g., licofelone) were developed but did not enter into the clinic due to safety concerns (Figure 2). Therefore, targeting mPGES-1/5-LOX dual inhibitors is an effective and safer strategy. Recently, mPGES-1/5-LOX inhibitor LFA-9 was designed and developed as a next-generation anti-inflammatory agent using a rational drug design strategy and exploring its pre-clinical anti-inflammatory efficacy [138] (Figure 2). ODC, FASN, and HMG COA-reductase are growth-factor/metabolic regulators, respectively, which are promising targets in obesity-mediated CRC. Statins are widely evaluated in pre-clinical and clinical levels for CRC prevention.

Over the last decade, several biomarkers/targets are identified and well characterized, and some others are under evaluation at the pre-clinical and clinical level, which have diagnostic and preventive/therapeutic importance in the precision chemoprevention of CRC. In addition, current and future efforts should focus on the identification, validation, and implementation of novel molecular biomarkers, which will achieve surrogate end point status. In turn, this may be helpful to the development of molecular targeted precision medicine with great promise in CRC prevention and treatment.

## Figures and Tables

**Table 1:** Expression status of molecular targets at different stages from initiation to progression of colorectal cancer (CRC).

	Normal	ACF	Adenoma	Adenocarcinoma	References
**Mutant/Dysregulated Signaling**					
*APC*					
Pre-clinical	+	−	−	−	[6]
Clinical	+	−	−	−	[3,4,5]
β-catenin					
Pre-clinical	+	++	++++	+++++	[7,8]
Clinical	+	++	++++	+++++	[6]
*TP53*					
Pre-clinical	+	+	−	−	[24]
Clinical	+	+	−	−	[20,21,22,23]
*KRAS*					
Pre-clinical	−	?	+++	++++	[12,13]
Clinical	−	+	+++	++++	[12]
*AKT*					
Pre-clinical	−	?	+++	+++++	[30]
Clinical	−	?	+++	+++++	[29]
*SMAD4*					
Pre-clinical	+	?	+	−	[19]
Clinical	+	?	+	−	[19]
**Inflammatory**					
COX-2					
Pre-clinical	−	+ *	++++	+++++	[34]
Clinical		?	++++	+++++	[32,35]
mPGES-1					
Pre-clinical	−	?	++++	+++++	[45,46]
Clinical	−	?	++++	+++++	[47,48]
5-LOX					
Pre-clinical	−	?	+++	++++	[49]
Clinical	−	?	+++	++++	[50,51]
iNOS					
Pre-clinical	−	+ *	+++	++++	[52]
Clinical	−	?	+++	++++	[53,54]
15-PGDH					
Pre-clinical	+++	?	−	−	[55]
Clinical	+++	?	−	−	[56]
STAT-3					
Pre-clinical	+	?	+++	+++++	[57]
Clinical	+	?	+++	+++++	[58]
Prostaglandin I_2_ Synthase	++	−	−	−	
Pre-clinical	++	−	−	−	[59,60]
Clinical	++	−	−	−	[61]
15-LOX					
Pre-clinical	++	?	−	−	[62,63]
Clinical	++	?	−	−	[64]
**Growth and metabolism**					
Ornithine decarboxylase					
Pre-clinical	+	?	++++	+++++	[65]
Clinical	+	?	++++	+++++	[66,67,68]
HMG Co-A-Reductase					
Pre-clinical	+	?	++++	+++++	[69,70]
Clinical	+	?	++++	+++++	[71]
Fatty acid synthase					
Pre-clinical	+	+	+++	++++	[72]
Clinical	+	+	+++	++++	[72,73]

“−” less expression or mutation; “+, ++, +++, ++++, +++++” overexpression or upregulation; “?” unknown expression levels; * Minimally expressed in ACF.

## Abbreviations

CRC	Colorectal cancer
ACF	Aberrant crypt foci
*APC*	*Adenomatous polyposis coli*
*KRAS*	*V-Ki-ras2 Kirsten rat sarcoma viral oncogene homolog*
COX-2	Cyclooxygenase-2
LOX	Lipoxygenase
FASN	Fatty acid synthase
ODC	Ornithine decarboxylase
iNOS	Inducible nitric oxide synthase
EGFR	Epidermal growth factor receptor
HMG Co-A-R	3-hydroxy-3-methyl-glutaryl-coenzyme A reductase
FASN	Fatty acid synthase
TGF-β1	Transforming growth factor-β1
15-PGDH	15-hydroxyprostaglandin dehydrogenase
STAT	Signal transducer and activator of transcription
NSAIDs	Nonsteroidal anti-inflammatory drugs
AOM	Azoxymethane
FAP	Familial adenomatous polyposis
CCR5	Chemokine receptor 5
PGI_2_	Prostacyclin
PGE_2_	Prostaglandin E_2_
mPGES-1	Microsomal prostaglandin E synthase-1
MDF	Mucin depleted foci
COXibs	COX-2 inhibitors
CCR5	Chemokine receptor 5
